# How polymer structure affects secondary cell wall patterns: A look at transdifferentiating protoplasts

**DOI:** 10.1093/plcell/koae238

**Published:** 2024-08-21

**Authors:** Arpita Yadav

**Affiliations:** Assistant Features Editor, The Plant Cell, American Society of Plant Biologists; Department of Biology, The Pennsylvania State University, University Park, PA 16802, USA

Plant cells are enveloped by cell walls, which regulate cell growth and provide structural support to the plant's form, ultimately dictating the physical characteristics and organization of plants. Plant cell walls are subdivided into 2 main types: primary cell walls and secondary cell walls (SCWs). The primary cell wall permits cell growth and is relatively pliable, but the SCW, which is thicker, gives the cell more strength and stiffness. Xylem tracheary elements (TEs), which move water and minerals from roots to shoots, are characterized by thickened SCWs that are enriched in cellulose, hemicelluloses like glucuronoxylan (in eudicots), and lignin.

In a groundbreaking study, **Sarah A. Pfaff and colleagues (Pfaff et al. 2024)** developed an innovative method to induce the transdifferentiation of Arabidopsis protoplasts into TEs by overexpressing *VASCULAR NAC DOMAIN* (*VND7*), which encodes a master transcription factor involved in the differentiation of xylem vessels (Yamaguchi et al. 2010). This system allowed the authors to observe and manipulate active SCW synthesis in protoplasts isolated from Arabidopsis T-DNA insertion lines, avoiding the need for complex breeding techniques. The authors were thus able to explore how cell wall polymers influence SCW patterning. By triggering TE formation in protoplasts isolated from mutants that exhibit faulty cellulose or xylan synthesis, the team characterized the resulting SCW banding patterns by observing cellulose with Calcofluor white staining and CBM3-A488 labeling, and xylan with LM10, a monoclonal antibody. The findings suggested that both polymers are required to generate distinct SCW patterns.

Precise integration of each cell wall polymer is crucial for the strength and utility of xylem SCWs. An irregular xylem *(irx*) phenotype, defined by collapsed xylem TEs, can be caused by various defects, including a disruption in cellulose synthesis, an error in xylan assembly (shortened backbone or altered side-chain decorations), or changes in lignin chemistry. The genes *IRX9* and *IRX14* encode family glycosyltransferases (GTs) that are essential for elongating xylan backbones (Brown et al. 2007). Mutations in these genes lead to GT-deficient plants displaying a collapsed TE phenotype and accumulate xylan with abnormally short chains. To examine the impact of xylan on SCW formation and assess cellulose-xylan interaction, Pfaff et al. observed the SCW patterns in transdifferentiating xylan biosynthesis mutants (*irx14*, *irx9*, *esk1*) as well as a cellulose synthase (CESA7) mutant (*irx3-4*) protoplasts that were transformed with *VND7* overexpression. The arrangement of cellulose bands in TEs was influenced by modified xylan. The proportion of cells with altered SCWs increased in xylan mutant protoplasts, with either shortened backbone structures or modification of the side chain attachments. Observations of these mutants showed that the altered xylan structure influenced TE cellulose banding patterns but did not entirely eradicate them (Fig.) Unique cell wall patterns in the cellulose synthase mutant *irx3-4* TEs suggested that cellulose deposition is necessary to form banded SCWs. Golgi and actin localization surrounding tightly packed chloroplasts likely caused the meshed pattern in *irx3-4*, which is not normally observed, resulting from immobile xylan deposition in cell wall domains that resembled the intracellular environment.

**Figure.**
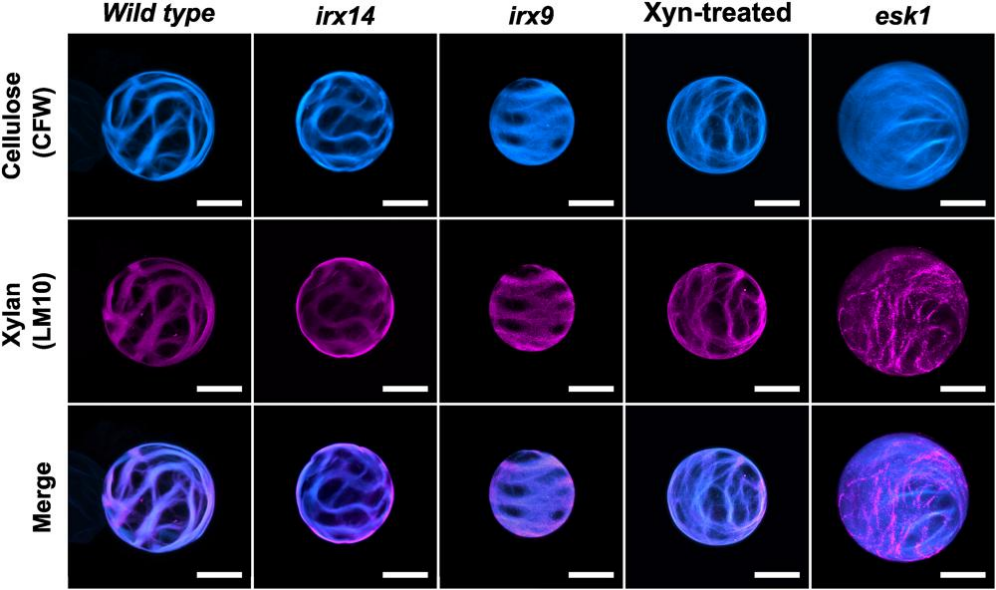
Cellulose (CFW, cyan) and xylan (LM10, magenta) patterning in transdifferentiating TEs with altered xylan in xylan biosynthetic mutants (*irx14*, *irx9*, *esk1*) and with xylanase (Xyn) treatment. The bottom row displays the superimposition of cellulose and xylan patterns in each protoplast sample. Scale bar −20 *μ*m. Reprinted from Pfaff et al. (2024), Figure 3F.

These findings highlight the pivotal role of a well-constructed cellulose-xylan network in orchestrating intracellular architecture to generate banded SCW domains. After SCW synthesis was complete, removing xylan did not impact banding patterns. The structure of the xylan polymer and the interactions between cellulose and xylan are therefore critical for developing distinct SCW banding patterns during active cell wall synthesis. The work of Pfaff et al. reveals that a correctly assembled cell wall network may assist in directing polymer deposition into distinctly banded domains. By detailing the transdifferentiation of protoplasts into TEs, the authors introduced a method for examining structured SCW synthesis in a tissue-free environment across various mutant backgrounds. They exploit the ability of protoplasts to be transiently transformed after isolation to introduce *VND7* overexpression in various SCW biosynthesis mutants. This attribute, coupled with the ease of imaging isolated cells, provides a new way to investigate the effect of polymer structure on cell wall synthesis. The method opens up a plethora of possibilities for studying cellular responses to transcriptional master regulators, which may lead to novel findings beyond the field of cell wall biology.

## Dive Curated Terms

The following phenotypic, genotypic, and functional terms are of significance to the work described in this paper:

plant cell AmiGo: PO:0009002

CESA7 Gramene: AT5G17420
CESA7 Araport: AT5G17420

NAC CHEBI: CHEBI:7421

xylem AmiGo: PO:0005352

ESK1 Gramene: AT3G55990
ESK1 Araport: AT3G55990

IRX14 Gramene: AT4G36890
IRX14 Araport: AT4G36890

IRX9 Gramene: AT2G37090
IRX9 Araport: AT2G37090

## References

[koae238-B1] Brown DM , Goubet F, Wong VW, Goodacre R, Stephens E, Dupree P, Turner SR. Comparison of five xylan synthesis mutants reveals new insight into the mechanisms of xylan synthesis. Plant J. 2007:52(6):1154–1168. 10.1111/j.1365-313X.2007.03307 17944810

[koae238-B2] Pfaff SA , Wagner ER, Cosgrove DJ. The structure and interaction of polymers affects secondary cell wall banding patterns in Arabidopsis. Plant Cell. 2024:36(10):4309–4322. 10.1093/plcell/koae233 PMC11449099 39163271

[koae238-B3] Yamaguchi M , Goué N, Igarashi H, Ohtani M, Nakano Y, Mortimer JC, Nishikubo N, Kubo M, Katayama Y, Kakegawa K, et al VASCULAR-RELATED NAC-DOMAIN6 and VASCULAR-RELATED NAC-DOMAIN7 effectively induce transdifferentiation into xylem vessel elements under control of an induction system. Plant Physiol. 2010:153(3):906–914. 10.1104/pp.110.154013 20488898 PMC2899931

